# Is There an Ideal Diet? Some Insights from the POUNDS Lost Study

**DOI:** 10.3390/nu16142358

**Published:** 2024-07-20

**Authors:** George A. Bray, Lu Qi, Frank M. Sacks

**Affiliations:** 1 Department of Clinical Obesity, Pennington Biomedical Research Center, Louisiana State University, Baton Rouge, LA 70808, USA; 2 Department of Epidemiology, School of Public Health and Tropical Medicine, Tulane University, New Orlean, LA 70112, USA; lqi1@tulane.edu; 3 Department of Nutrition, Harvard T.H. Chan School of Public Health, Boston, MA 02115, USA; fsacks@hsph.harvard.edu

**Keywords:** low carbohydrate, high protein, high fat, fasting, weight loss, gelatin diets, variability of weight loss, metabolic, behavioral and dietary factors

## Abstract

Diets for weight loss have a long history but an ideal one has not yet been clearly identified. To compare low-fat and lower carbohydrate diets, we designed The Preventing Overweight by Novel Dietary Strategies (POUNDS) Lost study. This is a 2 × 2 factorial study with diets of 20% or 40% fat and 15% or 25% protein with a graded carbohydrate intake of 35, 45, 55 and 65%. Weight loss, overall, was modest at nearly 6% with all four diets, and no significant dietary difference. The variability in weight loss in each diet group was significant, ranging from greater than 20% to a small weight gain. Studies of genetic variations in relation to weight loss showed that the diet that was selected could significantly affect weight loss, emphasizing that there is no ideal diet and more than one diet can be used to treat obesity. Weight loss was also influenced by the level of baseline triiodothyronine or thyroxine, and baseline carbohydrate and insulin resistance. Achieving a stable Health Eating Food Diversity Index, eating more protein, eating more fiber, engaging in more physical activity, sleeping better and eating less ultra-processed foods were beneficial strategies for weight loss in this trial. Although there is no “ideal diet”, both the DASH diet and the Mediterranean diet have clinical trials showing their significant benefit for cardiovascular risk factors. Finally, the lesson of the “Last Chance Diet”, which recommended a diet with protein from gelatin, proved that some diets could be hazardous.

## 1. Introduction

Dietary interventions for weight loss have been part of the history of medicine since its earliest days [1]. Several low-calorie diets varying in macronutrients have captured a lot of public interest, including high-protein diets, low-carbohydrate diets and low-fat diets [2,3]. To develop first-hand insight into diets and weight loss, the authors of this paper have designed and executed a study called Preventing Overweight Using Novel Dietary Strategies (POUNDS) Lost, which is one of the largest and longest weight-loss intervention trials [4]. We provide our insights into whether there is an ideal diet based on these findings from POUNDS Lost supplemented by other studies. We asked whether one of these diets would result in significantly more weight loss than the other, and if so, which one, and if not, what insights could we gain into the factors affecting dietary-induced weight loss.

## 2. The First Highly Popular Diet—The Banting Diet

The “Father of Block Buster Diets” was William Banting who published his diet in 1863 and introduced a lower carbohydrate diet to the public and for which he received international recognition [5,6]. William Banting was an undertaker to the Royal Family in the UK. As he became older, he found that he had to go down stairs backwards because he could not see his feet over his enlarged belly. He sought the advice of his physician William Harvey [7], not to be confused with the William Harvey who discovered the circulation of blood 150 years earlier [8]. Dr. Harvey had become aware of Claude Bernard’s research in Paris on the release of glucose from the liver and on this basis, he recommended reducing the intake of carbohydrates in the diet. The diet that Mr. Banting followed is shown in Table 1.

Mr. Banting was so elated by his loss of weight from just over 200 pounds to 160 pounds over the year he adhered to the diet that he self-published 1000 copies of a pamphlet in 1863 titled “Letter on Corpulence Addressed to the Public” [5]. This was followed by many subsequent editions on both sides of the Atlantic and translations into many languages. There were even conferences dedicated to the “Banting” diet [6]. The last edition that we could find of this book was published in 1902—40 years after the original for a very long press run for a popular diet book.

## 3. The POUNDS (Preventing Overweight Using Novel Dietary Strategies) Lost Study

### 3.1. Description of the POUNDS Lost Study

As new diets appeared on the horizon, the question was which, if any, combination of macro- or micronutrients was to be preferred [2,3]. The differing data on the effectiveness of diets for weight loss led us to design a 2-year trial comparing two levels of dietary fat (20 vs. 40%) and two levels of protein (15% vs. 25%) in a 2 × 2 factorial design [9]. This combination of fat and protein provided four levels of carbohydrate intake varying from 35% to 65%. All 811 of the participants were overweight or obese ([BMI] ≥ 25 kg/M^2^ and ≤40 kg/M^2^). Women composed 64% of the sample and were 30 to 70 years of age [51 + 9 years (mean + SD)] and 79% were White, 16% Black, and 4% Hispanic. People with diabetes or unstable cardiovascular disease, using medications that affect body weight, or with insufficient motivation as assessed by interview and questionnaire were ineligible to participate. There were two sites for the study, the Harvard T.H. Chan School of Public Health in Boston, and the Pennington Biomedical Research Center of Louisiana State University in Baton Rouge where the participants were randomly assigned to one of the four diets and participated in group activities and were encouraged to remain physically active. Over 80% of the participants completed the 2-year trial [10]. Body weight was measured at baseline 6, 12, 18 and 24 months of intervention. After 6 and 24 months, 24 h dietary recalls were collected in half of the participants from each site during telephone interviews on 3 nonconsecutive days. Calorie intake was restricted to 700 kcal/day below energy requirements, based on measurements of resting energy expenditure using a metabolic cart and multiplying it by an activity factor. All four diets were low in saturated fat and were in accordance with cardiovascular health guidelines. At both sites, a behavioral program with consistent content and intensity was implemented uniformly across the four groups. All participants signed consent forms approved by their respective institutions, and an external NIH advisory committee provided periodic assessment of the progress of the project.

A computer tracking system collected data on eight indicators of adherence throughout the first 6 months. Compliance with the diet was assessed over 3 days by 24 h dietary recalls conducted via telephone interviews at 6 and 24 months in a random 50% subset of participants at both sites. Individuals who achieved their assigned goals within a range of ±5% were labeled as “adherent”. A total of 241 participants wore pedometers at their belt line, aiming to promote adherence to the exercise program. The number of steps was recorded from this subset across each of the four dietary groups at baseline and 6 months. The prescription of a high-fat diet (vs. a low-fat diet) was found to be associated with higher levels of dietary adherence, although behavioral adherence was similar across the four intervention groups [10].

In POUNDS Lost, the participants lost an average of 6 kg in body weight at 6 months, but began to regain weight after 12 months. Such a weight change trajectory has also been observed in other long-term weight-loss trials. Among the 80% of the study population who completed the trial, the average weight loss was 4 kg. Analysis of the imputed data revealed consistent mean weight losses. Weight loss was 3.0 kg in the average-protein group, 3.6 kg in the high-protein group, and 3.3 kg in both diet groups assigned to the low- and high-fat diets at 2 years, respectively. The weight losses with the highest and lowest carbohydrate were 2.9 vs. 3.4 kg over 2 years. Similarly, no difference was observed in weight loss or weight maintenance at 2 years in the intent to treat analysis. Significant variation in weight loss among participants was found within each dietary intervention group [10].

### 3.2. Variability of Weight Loss

**Figure 1:**
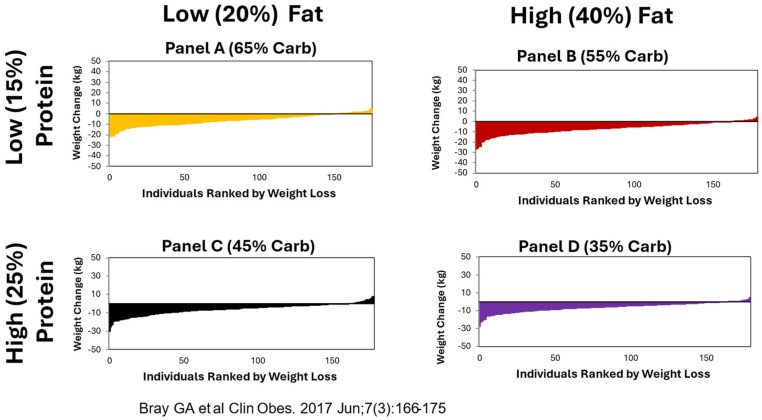
Similar patterns of weight loss with each diet over 6 months. Data from reference [11].

Although total weight loss was similar across all 4 diets at 6, 12, 18 and 24 months, there was considerable variability across each diet as shown at 6 months in Figure 1 [11]. Some people on each diet lost 20 kg or more, whereas at the other end, some gained weight. The large number of patients in each group and the variability within each diet offered the opportunity to explore many aspects of response to diet rather than assuming that no matter what diet was prescribed, weight loss would be similar.

## 4. Factors Affecting Weight Loss

### 4.1. Genes Interact with Diet

As the wealth of detailed data became clear, Dr Lu Qi at Harvard began to collaborate with us on analyzing the data, which has so far included over 70 publications. The first approach was to examine the interaction of single genes with diets. The genes were selected because they had been selected as potential candidates in genome-wide association studies (GWASs). Several genes were found to modify weight loss or decrease in fat mass or modify fat distribution in response to diet. These are summarized in Table 2.

The insulin receptor substrate-1 gene (*IRS1*) was the first gene that we explored. At 6 months, individuals in the highest carbohydrate group (65%) who had the risk-conferring CC genotype (rs2943641) had greater weight loss (*p* < 0.058), a larger decrease in circulating insulin (*p* = 0.009), and more insulin resistance measured by HOMA-IR (*p* = 0.015) than those with either the CT or TT genotype. This genotype effect on weight loss diminished after adjustment for changes in fasting insulin or HOMA-IR, suggesting that improvement in insulin resistance may influence weight loss. Our results thus indicated that participants with the *IRS1* rs2943641 CC genotype might obtain more benefits from weight loss and improvement of insulin resistance when eating a high-carbohydrate/low-fat diet than those without this genotype [12].

The second gene we tested was the glucose-dependent insulinotropic polypeptide receptor gene (*GIPR*), which may link overnutrition to obesity, insulin resistance, and type 2 diabetes. After 6 months on the test diets, those with the T allele of *GIPR* (rs2287019) assigned to the low-fat diet lost more weight (*p* = 0.06) and had greater decreases in fasting glucose (*p* = 0.006), fasting insulin (*p* = 0.03), and insulin resistance measured by HOMA-IR (*p* = 0.01) than those eating the high-fat diet where there were no significant genotype effects on changes weight, insulin levels or insulin resistance [13].

Dietary protein levels also interacted with some genes, including the *FTO* gene (rs1558902) (FTO = Fat Mass and Obesity-Associated Gene superfamily of hydroxy alpha-ketoglutarate-dependent hydroxylase). The risk A allele of the *FTO* genotype influenced not only body weight, but also body fat distribution as measured by dual X-ray absorptiometry (DXA) and computer aided tomographic (CT) scanning. We found that the risk allele (A) was significantly associated with a 1.51 kg greater weight loss in those assigned to the high-protein group (*p* = 0.010), but not in the low-protein group, by the end of the 2-year intervention. Consistent with the observations of change in body weight, we found that the risk allele (A) was associated with a greater loss of total fat, fat-free mass (FFM), % fat mass (FM%), and percentage of trunk fat at 2 years in the high-protein group, but not in the low-protein group. Interestingly, individuals with the T Allele had a greater reduction in fat-free mass (FFM) when eating the low-protein diet than those eating the high-protein diet. There was no interaction of *FTO* with the levels of dietary fat [14].

Response to the fat diets was affected by interaction with the *PPM1K* gene (rs1440581) (*PPM1K* = protein phosphatase 1K mitochondrial). This is a gene that is involved in the ratio of branched chain amino acids (BCAAs) between the liver and plasma. In those eating the high-fat diet, there was a weight loss recorded of nearly 8 kg at 12 months in those with the TT allele compared with about 6 kg in the TA allele and only 4 kg in the AA allele. In contrast, those eating the low-fat diet only had a small gradation of only 1 kg across the three alleles [15].

Carbohydrate diets interacted with the *FGF21* gene. The effects of the CC, CT and TT genotypes differed significantly between low- and high-carbohydrate diets. Those eating the low-carbohydrate diet who had the TT genotype lost about 4% of body fat, those with the CT genotype lost 2% and those with the CC genotype lost just under 1% of body fat. In contrast, those eating the high-carbohydrate diet showed the reverse pattern, as those with the TT genotype lost just over 2% body fat, those with the CT genotype lost just over 3% and the individuals with the CC genotype lost over 5% of body fat. Thus, the response to the popular low-carbohydrate diet may depend on which genes are expressed by *FGF21* [16].

Variants in the *TCF7L2* gene have been associated with increased risk for type 2 diabetes as well as increased adiposity. In POUNDS Lost, we investigated the effect of two polymorphisms of *TCF7L2* (rs7903146 and rs12255372) on adiposity and glucose levels in the four diet groups. We found that those with the TT allele of TCF7L2 (rs12255372) eating the low-fat diet lost significantly more body weight and had larger decreases in percent fat mass and percent trunk fat than either of the other genotypes [17].

*NPY* genetic variants interacted with the impact of dietary fat intake on waist circumference and total adipose tissue, visceral adipose tissue and subcutaneous adipose tissue. After two years of the high-fat diet, those with the CC genotype had the greatest decrease in each measurement. Those with the CT genotype had a lower decrease and the final group with the TT genotype showed consistent increases in all three fat compartments and a smaller decrease in the waist circumference than with the other two genotypes. In contrast, those eating the low-fat diet had the greatest decrease if they had the TT genotype. The rs16147 T allele thus appeared to be associated with more adverse changes in the abdominal fat deposition with the high-fat diet group than with the low-fat diet group [18].

DNA-methylation may also influence weight loss for different diets. Among the participants assigned to the average-protein (15%) diet, those with the lowest tertile of baseline regional DNA methylation at *ABCG1* (ATP-binding cassette sub-family G member) had greater reductions in visceral adipose tissue (VAT), subcutaneous adipose tissue (SAT), deep-subcutaneous adipose tissue (dSAT), and total adipose tissue (TAT) measured by computed tomographic scanning (CT) consistently from baseline to 2 years, whereas those with higher DNA methylation at baseline in *ABCG1* showed slight reductions in abdominal fat distribution at 6 months, which they then regained from 6 months to 2 years [19].

For the *NFATC2IP* gene (Nuclear Factor of Activated T Cells 2 Interacting Protein), we determined genetic (rs11150675) and transcriptional (ILMN_1725441) variations, as well as cis-DNA methylation at cg26663590. In response to the high-fat diet, carrying the A allele was marginally associated with a greater decrease in weight. In contrast, the low-fat diet produced a significantly smaller decrease in weight. In those eating the high-fat diet, the baseline methylation at cg26663590 mediated an estimated 52.8% of the effect of the *NFATC2IP* rs11150675 genotype on weight change over 2 years. In contrast, there was no such mediation in those eating the low-fat group. Finally, carrying the rs11150675 A allele was associated with a lower methylation level at cg26663590 [20].

### 4.2. Combining Genes in Genetic Risk Scores for Improved Prediction

Using data from genome-wide association studies, a GRS with 20 single nucleotide polymorphisms (SNPs) for gut microbial taxa was obtained (Table 2). A higher GRS for the relative abundance of gut microbial taxa was significantly associated with greater reductions in waist circumference, total fat mass (FM), whole-body total percentage of fat mass (FM%), and percentage of trunk fat (TF%) at 2 years. In the average-protein diet group, a higher GRS for microbial abundance GRS was associated with greater reductions in total fat mass (FM), %FM, and % total fat at 6 months, while no associations were found in the high-protein diet group [22].

A GRS for *AMY1* was calculated using nine single nucleotide polymorphisms (SNPs) associated with copy number variation in *AMY1* gene loci, with higher AMY1-GRS indicating higher activity of salivary amylase. In response to the 35% low-carbohydrate diet, those with the higher GRS for *AMY1* were associated with greater decreases in weight and waist circumference. A low-carbohydrate diet interacted with the impact of carbohydrates on BMI and waist [23].

### 4.3. Metabolic and Behavioral Changes

Several metabolic factors and behavioral changes were associated with weight loss in the POUNDS Lost study. Many of them are summarized in Table 3.

Analysis of the early effects of food intake was facilitated by the computer tracking system that was used during the first 6 months to collect data on eight indicators of adherence to the four diets [31]. The two factors among these eight that accounted for 66% of the variance were (1) behavioral adherence and (2) dietary adherence. Behavioral adherence did not differ across the four diet groups, but prescription of a high-fat diet as opposed to a low-fat diet was associated with higher levels of dietary adherence [32]. Early behavioral adherence was associated with changes in percent weight loss and waist circumference at 6 months and again at 24 months, but, interestingly, it was not associated with cardiovascular disease risk factor levels [33]. These data from the computer tracking system were also used in a computer program to predict future responses. Using computer modeling, Thomas and colleagues [34] developed ways to predict the likelihood of achieving a >5% weight loss at 1 year based on changes during the first 3 months that can be of value in this age personalized medicine.

Adherence to behavioral programs is well known to correlate with better weight loss. This was shown in the POUNDS Lost study where over 2 years, every session attended resulted in 0.2 kg of weight loss with no difference across dietary groups. The more sessions an individual attended, the greater their weight loss [10].

We analyzed circulation thyroid hormones and found that elevated levels of baseline free T3 and free T4 were associated with greater 6-month weight loss (Table 3) [25].

Baseline glucose and insulin predicted the response to diet in several studies [35,36]. Participants with normal plasma glucose (FPG < 100 mg/dL) in the POUNDS Lost study lost 2.6 kg more body weight on the high-protein/low-fat diet than when assigned to the average-protein/low-fat diet (n = 136). Those with more insulin resistance (≥4 as measured by HOMA-IR) lost 3.6 kg more body weight than those assigned to the high-fat/high-protein (n = 35) diet compared to the high-fat/average-protein diet (n = 33) [37].

Weight change is primarily regulated by the balance of energy intake and expenditure and intervention-induced alterations in appetite play a key role in modulating energy intakes. In a study, we assessed a group of factors defining appetite and found that the food craving score for high-fat foods was related to a decrease in energy intake and more weight loss. In addition, greater cognitive restraint of eating was associated with less weight loss and weight regain [38].

Using individuals who had their food intake recorded at baseline, 6 and 24 months by dietary recall, Apolzan et al. examined food cravings using the food craving inventory (FCI) and actual foods eaten [39]. There was an association between the change in food craving inventory items consumed from baseline and change in cravings at months 6 and again at 24 months. However, there was no association between the change in the amount of energy consumed per FCI item from baseline and change in cravings [40].

Genetic factors might be expected to modify appetitive behaviors, especially those involving melanoortin-4 receptor (*MC4R*). This was revealed after it was found that the low-protein diet with 15% of energy from protein compared with 25% of energy, respectively, significantly modified *MC4R* genetic effects on changes in appetite score and craving at 2 y, after adjustment for age, sex, ethnicity, baseline BMI, weight change, and baseline perspective phenotype (Table 2) [24]. The obesity-predisposing A allele was associated with a greater increase in overall appetite score and craving compared with the non-A allele among participants who consumed a high-protein diet. The *MC4R* genotype did not modify the effects of fat or carbohydrate intake on appetite measures. Our data suggest that individuals with the *MC4R* rs7227255 A allele rather than the non-A allele might experience greater increases in appetite and food craving when consuming a high-protein weight-loss diet [21].

A genetic risk score for lean body mass (LBM) was also used to assess its association with changes in appetite (Table 2). A lower GRS for LBM (indicating a greater genetic predisposition to LBM) was associated with greater decreases in the total appetite score, hunger, fullness, and prospective consumption in participants in the low-fat diet group, whereas no significant associations with these appetitive measures were observed in the high-fat diet group. In addition, lower GRS for LBM was associated with a greater reduction in body weight and waist circumference among participants assigned to the low-fat diet group, whereas no associations were observed in the high-fat diet group. These interactions were attenuated, along with weight regain, from 6 months to 2 years [24].

The amounts of physical activity were measured by a pedometer in a subset of participants. Xue et al. led a study to investigate whether changes in physical activity during dietary interventions were associated with changes in body fat distribution. It was found that an increase in physical activity measured by a pedometer was associated with a greater reduction in body weight, waist circumference and body fat distribution, including visceral fat (Table 3) [26].

### 4.4. Nutrient-Related Changes Alter Response to Test Diets

A number of different dietary changes had a significant influence on the amounts of weight lost, including the selection of a larger variety of healthful foods, the amount of dietary protein ingested, the amount of fiber eaten, and the intake of ultra-processed foods.

The variety of foods eaten was assessed with the US Healthy Food Diversity Score in the subset of participants who had records of their food intake by telephone interview. At 6 months, those whose index increased lost significantly more weight and waist circumference than the other two groups. There were also suggestive changes in total body fat and trunk fat [27].

Increasing intake of dietary protein was also associated with more weight loss as quantitated by the measurement of the ratio of urinary nitrogen to creatinine excretion (Table 3). The lowest quartile lost about 2.5 kg compared to 7.5 kg for the highest quartile [11].

Finally, the selection of unprocessed foods was associated with more weight loss. Using the consumption of Nova 1 + 2 foods, the least ultra-processed foods, as the dividing line, those with the highest consumption of ultra-processed foods lost 5.3 kg compared to 8.3 kg in the highest tertile who were eating lower levels of highly processed foods [28].

Disturbed sleep has been linked to the development of obesity. To test this idea, we measured sleep disturbance in the POUNDS Lost trial. In our study, we found that greater sleep disturbance was indeed associated with up to a 3-fold higher risk of failure to lose weight during the 6-month weight loss phase of the study [29].

Those whose fiber intake was in the upper quartile of change from baseline lost just over 10 kgs, compared to 6 kgs for the lower quartiles (Table 3) [30].

## 5. Discussion

The answer to the question of whether there is “an ideal diet” is clearly no. This has been captured in the review by Chao et al. when they stated that “There is not a one-size-fits-all diet for obesity treatment” [41].

One of the striking findings from the POUNDS Lost study was the variability in weight loss with each of the four diets and the number of factors that influenced this weight loss. This variability is present in all studies of weight loss and generally dwarfs the difference in mean weight loss between diets [42]. Although this might be interpreted to mean all diets are similar and it is the patient that differs, the data from POUNDS Lost show that both genetic and non-genetic factors make significant differences in how individuals respond to different diets. The conclusion from this is that there is a need for a variety of diets to help people wanting to lose weight. The best diet is one which the patient can adhere to most easily and which produces the weight loss they want.

Diets have been evaluated from several different perspectives. First are the results of expert opinions. Next are the reviews and metanalyses that have been carried out to compare one diet with another.

### 5.1. Expert Opinions

For the last several years, the US News and World Report has empaneled a group of experts and asked them to evaluate which of the current diets ranks best overall as well as in several subcategories [43]. The overall ranking from 2023 has been summarized in Table 4 along with the scores and subdivided by diets with higher and lower scores. The top two diets are the Mediterranean diet and the DASH diet, each of which has published data showing clinical benefits for cardiovascular diseases [44,45]. Indeed, it was the development of the DASH diet that began the collaboration of two of the current authors (GB and FS).

The DASH Diet focuses on fruits, vegetables, and low-fat dairy and has been shown in clinical trials to reduce blood pressure significantly [44]. The other diet is the Mediterranean-style diet that focuses on legumes, fish, poultry nuts, and wine with meals. In the PREDIMED study of the Mediterranean diet, CVD mortality was significantly reduced by 29% [45].

### 5.2. Comparison of Diets

As noted earlier, since the time of Banting’s successful diet, there has been a never ending run of diet books that include one or more of the following: very low-calorie diets, usually considered between 200 and 800 kcal/d; low-calorie diets with 800 to 1500 kcal/d; portion-controlled diets using commercially available products; low-carbohydrate diets; low-fat diets; high-protein diets; and diets that use timed eating strategies often restricting meals on two of the seven days per week. Chao et al. [41] have published a recent narrative and analytical review of diets used for weight loss in patients with obesity. It is clear from their review that all successful approaches must reduce calorie intake relative to expenditure. In their comparison of very low-energy diets (VLEDs) to low-energy diets (LEDs) with more calories, Chao et al. [41] found that the individuals adhering to the VLED lost more weight. They then provide a detailed discussion of meta-analyses comparing other dietary regimes and conclude that “There is not convincing evidence that one diet is universally easier to adhere to than another for extended periods, a feature necessary for long-term weight management”. For a more detailed discussion of dietary approaches, this is an excellent review whose length is beyond the scope of this paper.

### 5.3. Adverse Effects of Some Diets

The success of the Banting diet in the last part of the 19th century led to an ongoing list of diet books designed to “cure” obesity often with special secrets [46,47,48,49]. One unfortunate outcome was the gelatin-based diet published by Linn and Stuart [50]. Even though a commission at the time of the French Revolution had shown that gelatin-based diets were incompatible with long life in animals, Linn and Stuart published the “Last Chance Diet” using a gelatin-based formula [50]. Of the more than 50 cases reported to the FDA, they investigated 17 individuals who died suddenly of an unusual ventricular arrhythmia called torsade de points [51]. The median duration of the diet was 5 months and consisted entirely or largely of protein, usually composed of gelatin. Factors common to all cases were marked obesity at the onset of dieting, prolonged use of extremely low-calorie diets usually with gelatin and approximately 300 to 400 kcal daily, and significant and rapid weight loss. The conclusion was to practice moderation in selecting diets.

## 6. Conclusions

Variability in response to diets is one of the key lessons from this analysis. The second key lesson is that many genetic and non-genetic factors can modify weight loss with any diet. Thus, a variety of diets have a valuable place in the management of people with obesity. Finally, some diets can be hazardous to your health.

## Figures and Tables

**Table 1:** Mr Banting’s diet [8].

Components of Intake	Average	% as Calories
Energy Intake	1714 kcal/d	
Protein intake	115 g/d	27%
Fat Intake	42 g/d	22%
Carbohydrate Intake	119 g/d	28%
Alcohol	56 g/d	23%

Data analysis performed by Dr. Catherine M. Champaign, PhD. With this diet, he ate 319 kcal at breakfast, 835 kcal at dinner, 240 kcal at tea time and 320 kal at supper. Breakfast: 150–180 g (5–6 oz) of meat or broiled fish (not a fat variety of either); a small biscuit or 30 g (1 oz) of dry toast; a large cup of tea or coffee without cream, milk or sugar. Dinner at 1 PM: Meat or fish as at breakfast, or any kind of game or poultry, same amount; any vegetable except those that grow underground, such as potatoes, parsnips, carrots, or beets; dry toast, 30 g (1 oz); cooked fruit without sugar; good claret, 300 cc (10 oz), Madiera or sherry. Tea at 5 PM: Cooked fruit, 60 to 90 g (2–3 oz); one or two pieces of zwieback; tea, 270 cc (9 oz), without milk, cream or sugar. Supper at 8 PM: Meat or fish, as at dinner, 90–120 cc (3–4 oz); claret or sherry, water, 210 cc (7 oz). Fluids: restricted to 1050 cc (35 oz) per day.

**Table 2:** Summary of gene interactions with diet in the POUNDS Lost Study.

Gane Name	Genotype or Phenotype	Diet with Most Weight or Fat Loss	Ref #
Insulin receptor substrate-1 gene (*IRS1*)	CC genotype (rs2943641) TC and TT	Highest carbohydrate diet at 6 months lowered weight, insulin, and insulin Res (HOMA-IR) at 6 months Lowest carbohydrate diet at 6 months lowered insulin and HOMA-IR but not weight	[12]
Glucose-dependent insulinotropic polypeptide receptor (*GIPR*)	T allele (rs2287019) Changes in glucose, insulin and HOMA-IR paralleled weight changes	Low-fat diet: TT lost 8 kg; CT lost 6 kg; CC lost 4.5 kg High-fat diet: TT lost 5.5 kg; CT lost 5.8 kg and CC lost 5.9 kg	[13]
Fat Mass and Obesity-Associated Gene (*FTO*)	A allele (rs1558902) T allele	High-protein diet associated greater with loss; Low-protein diet associated with loss of fat free mass (FFM); greater loss of TAT, VAT and SAT	[14]
Protein phosphatase 1K mitochondrial (*PPM1K*)	T allele (rs1440581)	High-fat diet: TT allele lost 8 kg at 12 mos; the CT allele 6 kg and the CC allele 4+ kg	[15]
Fibroblast Growth Factor-21 (*FGF21*)	Graded response across genotypes (rs838147): C Allele T alleles	High-carbohydrate diet: C allele lost most body fat; Low-carbohydrate diet: T Allele lost most body fat	[16]
Transcription factor 7-like 2 (*TCF7L2*)	TT (rs12255372)	Low-fat diet: TT genotype had more weight and fat loss than other genotypes	[17]
Neuropeptide Y *(NPY)*	C allele (rs16147) T allele	High-fat diet: greater fat loss with C allele Low-fat diet: greater fat change with T allele	[18]
Baseline DNA methylation at *ABCG1* (ATP-binding cassette sub-family G member 1)		Average-protein diet had significant reductions in body weight, waist circumference, TAT, VAT, SAT and dSAT at 6 months and 2 years High-protein diet did not have these effects	[19]
Nuclear Factor of Activated T Cells 2 Interacting Protein (*NFAT2CIP*)	*NFAT2CIP* (rs11150675) A allele Transcriptional variations (ILMN_1725441) cis-DNA methylation at (cg26663590)	Low-fat diet: non-A allele lost more wt; lower baseline for transcriptional variant ILMN lost more weight High-fat diet: no effect of A allele on wt; higher baseline ILMN resulted in more wt loss	[20]
Melanocortin-4-receptor-4 (*MC4R)*	*MC4R* (rs7227255) A allele	High-protein diet: obesity-predisposing A-allele had a greater increase in appetite score and craving at 2 years than non-A allele. No interaction with fat diets	[21]
GRS for the relative abundance of gut microbial taxa	20 SNPs	Average-protein diet reduced 6 mo fat mass High-protein diet: no effect	[22]
GRS for amylase 1 (*AMY1*)	9 SNPs	35% carbohydrate diet reduced weight and waist circumference 65% carbohydrate diet increased weight and waist circumference	[23]
GRS for low body mass index—(lower GRS associated with more LBM)	5 SNPs	Low-fat diet: lower GRS for LBM resulted in a greater decrease in body weight, waist circumference, and in appetite, hunger, fullness, and prospective fullness High-fat diet: no interaction of GRS	[24]

**Table 3:** Some metabolic, dietary and behavioral variables.

Variable	Time	Tertiles ^1^						Comment	Ref #
		1		2		3			
Free-Triiodothyronine	6 mos	3.9 kg		4.6 kg		5.4 kg		Similar response for free-thyroxine; data from Model 1	[25]
Physical Activity	6 Mos	6.2 kg		7.0 kg		9.5 kg		Measured by pedometer	[26]
Healthy Dietary Variety	6 mos	6.6 kg		6.3 kg		8.2 kg		US Healthy Food Diversity index; change at 6 mos 1 = reduced; 2 = stable; 3 = increased variety	[27]
Ultra-Professed Food		5.3 kg		7.5 kg		8.3 kg		Nova 1 and 2 foods	[28]
		**Quartiles**							
		**1**	**2**		**3**		**4**		
Sleep Quality	6 mos	6.0 kg	4.8 kg		4.8 kg		2.0 kg	Degree of sleep disturbance: 1 = no disturbance; 2 = slight; 3 = moderate; 4 = severe	[29]
Protein	24 mos	2.6	5 kg		7.1 kg		7.5 kg	Measured as nitrogen excretion/g creatinine; quartiles of response	[11]
Fiber	6 mos	5.8 kg	5.8 kg		7 kg		10.3 kg		[30]

^1^ = Weight (kg) indicates weight loss in each tertile or quartile of the variable.

**Table 4:** Popular weight loss diets based on their rankng by the US News and World Report in 2023.

Higher Scored Diets	Score	Lower Scored Diets	Score
Mediterranean Diet	4.6	Zone	2.8
DASH Diet	4.4	Nutritarian	2.7
The Flexitarian Diet	4.4	Jenny Craig	2.5
MIND Diet	4.3	Nutrisystem	2.5
TLC Diet	4.1	South Beach Diet	2.5
Mayo Clinic Diet	4.0	Keyto Diet	2.5
Volumetrics Diet	4.0	Paleo Diet	2.3
Weight Watchers Diet	3.8	Keto Diet	1.9
Dr Weil’s Ant-inflammatory	3.7	Atkins Diet	1.8
Ornish Diet	3.6	Optavia Diet	1.8
Noom Diet	3.5	SlimFast Diet	1.8
Pritikin Diet	3.5	Raw Food Diet	1.7
